# Platelets Apoptosis and Clearance in The Presence of Sodium Octanoate during Storage of Platelet Concentrate at 4˚C

**DOI:** 10.22074/cellj.2020.6697

**Published:** 2019-10-14

**Authors:** Vahid Baghdadi, Fatemeh Yari, Mahin Nikougoftar, Mohammad Hessam Rafiee

**Affiliations:** Blood Transfusion Research Center, High Institute for Research and Education in Transfusion Medicine, Tehran, Iran

**Keywords:** Cold Temperature, HepG2, Octanoic Acid, Platelet

## Abstract

**Objective:**

Platelet (PLT) storage at 4˚C has several benefits, however, it is accompanied by increased clearance of PLTs after transfusion. In this study, we evaluated the potential of sodium octanoate (SO) for reducing apoptosis and clearance rate of PLTs after long-term storage in cold.

**Materials and Methods:**

In this experimental study, PLT concentrates (PCs) were stored for 5 days under the following three conditions: 20-24˚C, 4˚C, and 4˚C in the presence of SO. To measure the viability of PLTs, the water-soluble tetrazolium salt (WST-1) assay was performed. Phosphatidylserine (PS) exposure was determined on PLTs using flow cytometry technique. The amount of human active caspase-3 was determined in PLTs using an enzyme-linked immunosorbent assay. Additionally, the amount of PLT ingestion or clearance was determined by using HepG2 cell line.

**Results:**

The viability was higher in the SO-treated PLTs compared to the other groups. The level of PS exposure on PLTs was lower in the SO-treated PLTs compared to the other groups. The amount of active caspase-3 increased in all groups during 5-day storage. The highest increase in the amount of caspase-3 levels was observed at cold temperature. However, PLTs kept at 4˚C in the presence of SO had a lower amount of active caspase-3 compared to PLTs kept at 4˚C. The amount of PLTs removal by HepG2 cells was increased for 4˚C-kept PLTs but it was lower for PLTs kept at 4˚C in the presence of SO but, the differences were not significant (P>0.05).

**Conclusion:**

SO could partially moderate the effects of cold temperature on apoptosis and viability of platelets. It also decreases the ingestion rate of long-time refrigerated PLTs *in vitro*. Further studies using higher numbers of samples are required to demonstrate the effect of SO on reducing the clearance rate of PLTs.

## Introduction

Platelets (PLTs) are anuclear cell fragments that act primarily as homeostasis regulators, and they play a role in angiogenesis and innate immunity (1-3). Transfusion of PLT concentrate (PC) is accompanied by a high prevalence of anaphylactic and febrile reactions compared with fresh frozen plasma (FFP) and red blood cells (4). Storage of PC at room temperature leads to PLT storage lesion, which includes a series of harmful changes that negatively affect PLT function (5).

PCs are typically stored at 20-24˚C with continuous agitation. Storage temperature conditions limit PLTs storage time to 5-7 days due to increased risk of bacterial contamination (6). It was shown that from every 1,000 to 5,000 units of PC, a single unit is infected with bacteria (7). Due to insufficiencies in PCs supplies, an alternative strategy is needed. Over the years, researchers have provided alternative methods for increasing the half-life of PLTs, including the use of additive solutions like PLT additive solution (PAS), storage in cold, lyophilization, etc. Although PLT storage in cold can have several benefits, including reducing the rate of bacterial growth and increasing hemostatic activity, PLT storage in cold was obsolete in the 1970s due to increases in their clearance rates. Several *in vitro* studies since 1970 showed that maintaining PLTs at 4˚C results in better functional and metabolic responses, such as aggregation, adhesion to the subendothelium and minimum lactate formation. PLTs stored at 4˚C had a better function compared to PLTs kept at room temperature; by employing the former, the bleeding time reduced in patients with thrombocytopenia, volunteers receiving aspirin, and in patients with aplastic thrombocytopenia (8, 9).

Short-term maintenance of PLTs in cold causes clustering and exposure of β-N-acetylglucosamine residues that make them detectable by liver macrophages (10). In comparison, PLTs stored in cold for a long time have a severe increase in galactose exposure, specifically on glycoprotein Ibα (GPIbα). Galactose is a ligand for asialoglycoprotein receptors (ASGPRs), and ASGPRs mediate hepatocytes-induced clearance (11).

Studies have shown that some substances such as trehalose, significantly reduce PLT phagocytosis after transfusion, improve the activity of PLTs and retain the response of cold-stored PLTs to agonists *in vitro*; this could be related to the prevention and inhibition of PLTs’ apoptosis in cold (12). It has been indicated that activation of caspases leads to rapid removal of PLTs from the circulation (13). Active caspase-3 induces apoptosis by cleaving the vital proteins of the cell (14). According to the previous studies, the amount of caspase-3 is increased during PLT storage at room temperature (15- 18). Additionally, storage in cold increases the amount of caspases in the PLTs (12).

To overcome the problems that platelets encounter in the cold, this study was conducted. We evaluated the potential of sodium octanoate (SO) as a substance which is known as a protein stabilizer against heat. Octanoic acid is a saturated medium-chain fatty acid with an 8-carbon backbone. This substance is present at a concentration of 0.2 μM in serum (19). SO was used for providing infusible PLT membranes (IPMs), as a protein stabilizer against 20- hour heating at 60˚C used for deactivation of viruses. An increase in IPM binding to von willebrand factor (vWF) was observed at an optimum concentration of SO (20). It was shown that 800-1200 μM of octanoic acid has longterm negative effects on embryonic and fetal growth in a mouse model; however, it has no harmful effects on the growth of the embryo at ≤400 μM concentrations (19). Also, it was found that medium-chain fatty acids do not have a toxic effect on humans and animals (21). In this study, we aimed to use SO for reducing the problems related to cold storage of PLTs. For this purpose, we kept the PCs under the following conditions: 20-22˚C with agitation, 4˚C and 4˚C in the presence of SO without agitation. Subsequently, we analyzed the quality of PLTs during 5-day storage in terms of apoptosis rate and the clearance level, by using a cell line originated from human hepatocytes.

## Materials and Methods

### Preparation of platelet concentrates

This experimental study was approved by the Ethics College’s Bioethics Committee (IR. TMI.REC.1396.004). Twelve PC bags with citrate phosphate dextrose adenine anticoagulant solution (CPDA1) (Macopharma, France) were prepared from Iranian Blood Transfusion Organization (IBTO). PLT-rich plasma (PRP) method was used for the preparation of PCs.

Prior to SO addition to the bags, all the parameters of the study were evaluated on day 1. After evaluating the parameters, PCs were divided into three equal volumes (A, B, and C) using a digital balance (Sartorius, Germany) and a Terumo Sterile Connecting Device (TSCD- II, Terumo Tubing welder, Japan). SO was added to one of the bags and the bag was transferred to a 4˚C refrigerator, the second bag was also kept under the same condition and the third bag was stored at 20-24˚C in a shaker-incubator and agitated. It should be noted that PLTs were stored at 4˚C without agitation because according to a previous study, agitation does not improve the quality of PLTs stored at cold in comparison with PLTs stored at cold without agitation (22).

### Determination of the effective concentration of sodium octanoate

SO (Merck, Germany, Grade; Ph Eur, NF) with the chemical formula (CH_3_ (CH_2_)_6_COONa) was used. Different concentrations of SO (100, 200, 400, and 800 μM) were examined. The PLT bags were placed at 4˚C without agitation or at 22˚C with agitation for 5 days. PLT count, mean PLT volume and phosphatidylserine (PS) were examined on the storage days using an automated hematology analyzer (Sysmex XT-2000i, Kobe, Japan) (data not shown).

### Evaluation of the viability and metabolic activity of platelets using WST-1 assay

To measure the mitochondrial activity of PLTs, we used the WST-1 cell proliferation assay kit (WST-1, Cayman, USA). In this method, tetrazolium salt is changed to formazan in viable cells by cellular mitochondrial dehydrogenases. Here, PLTs were diluted with phosphate buffered saline (PBS) and 10×10⁶ PLTs (100 μl) were added into each well. Then, 10 μl of WST-1 blend solution was added to each well and the plate was incubated for 4 hours at 37˚C in a CO₂ incubator. The absorbance of the wells was measured using a microplate reader at 450 nm.

### Evaluation of phosphatidylserine exposure

The levels of PS exposure were determined on the surface of PLTs using Annexin V-FITC assay kit )Biolegend, US (and flow cytometry technique. In summary, a PLT count of 1.5×10^6^ cells was incubated in 300 μl of annexin V binding buffer. Then, 5 μl of FITC-labeled annexin V was added and the tubes were incubated at room temperature for 20 minutes. Samples were analyzed by flow cytometry technique using the CyFlowR Space (Partec, Germany).

### Human active caspase-3 evaluation

Human active Caspase-3 level was determined using an enzyme-linked immunosorbent assay kit (Invitrogen, US) with the sensitivity of 1.25 ng/ml. At first, we prepared a cell extraction buffer according to the manufacturer’s instructions. For preparation of the cells, we collected 5×10⁸ PLTs by centrifugation, and then we washed PLTs three times with PBS. We added the cell extraction buffer to the pellet and incubated them for 15 minutes. After centrifugation at 4000 g for 10 minutes, the supernatant was collected in a clean tube. ELISA assay was done according to the kit instructions. After completing the reactions, the optical density of the wells was read at 450 nm. Finally, the concentration of unknown samples and controls was determined using the plotted standard curve.

### Preparation of mepacrine-labeled platelets

 Mepacrine is a polyphenol mixture which has an emission wavelength within the range of FITC (Fluorescein). Based on the PLT count on the first day, 5×10^7^ cells were treated with 30 μl of phosphate buffered saline (PBS) and 20 μl of 20 mg/mL mepacrine and incubated for 30 minutes at ambient temperature in the dark. Afterward, cells were washed with PBS buffer three times by centrifugation at 1200 g for 15 minutes. Finally, PLTs were exposed to HepG2 cells.

### Ingestion of PLTs by HepG2 cells *in vitro*

Initially, cells of the human hepatocellular cancer cell line (HepG2) were cultured in DMEM-F12 supplemented with 10% fetal bovine serum (FBS). After the growth of the adherent cells, they were starved for 30 minutes in serum-free medium. Then, mepacrine-labeled PLTs (5×10^7^) were added to each well and incubated at 37˚C for 30 minutes. After the incubation time, the wells were washed three times with PBS. Subsequently, HepG2 cells were detached from the culture plates by treatment with trypsin at 37˚C for 10 minutes. The ingestion of mepacrinelabeled PLTs by HepG2 cells was evaluated by flow cytometry technique. Unbound PLTs were separated from HepG2 cells by their forward and scatter characteristics. HepG2 cells containing the ingested PLTs were identified by their green fluorescence and the HepG2 cells having the adherent but not containing PLTs were identified by labeling with PE-anti-CD42b.

### Statistical analysis

SPSS v.22.0 (IBM Corporation, US) software was used to analyze and process the data. For evaluation of the effects of each treatment at different time points, we used two-way repeated measure ANOVA with two withinsubject factors (3 paired group×3 times).

## Results

### The effective concentration of sodium octanoate

Due to the positive effects of SO on the evaluated factors of PLTs, the optimum concentration of 200 μM was selected. Among the parameters of study, at this concentration of SO, the counts of PLTs was higher, and the PS exposure was lower on the third and fifth days of storage in comparison to other concentrations. Additionally, based on WST-1 assay, higher viability of PLTs was observed at this concentration of SO (data not shown).

### Cell viability (WST-1) assay

The metabolic activity and survival rate of PLTs were decreased during 5-day storage. The lowest survival rate was detected for 22˚C-stored PLTs. The metabolic activity of PLTs was well-maintained in PLTs treated with SO (4˚C) in comparison to those that were only kept at 4˚C, but the differences were not statistically significant between the groups. The mean ± SD values for WST-1 [optical density 450 (OD_450_ nm)] were as follows: on day 1 of storage 0.522 ± 0.97, on day 3 (4˚C) 0.421 ± 0.56, on day 3 (4˚C+SO) 0.493 ± 0.73, on day 3 (22˚C) 0.274 ± 0.60, on day 5 (4˚C) 0.358 ± 0.55, on day 5 (4˚C+SO) 0.412 ± 0.56, and on day 5 (22˚C) 0.226 ± 0.67

### The exposure level of phosphatidylserine

**Fig 1:**
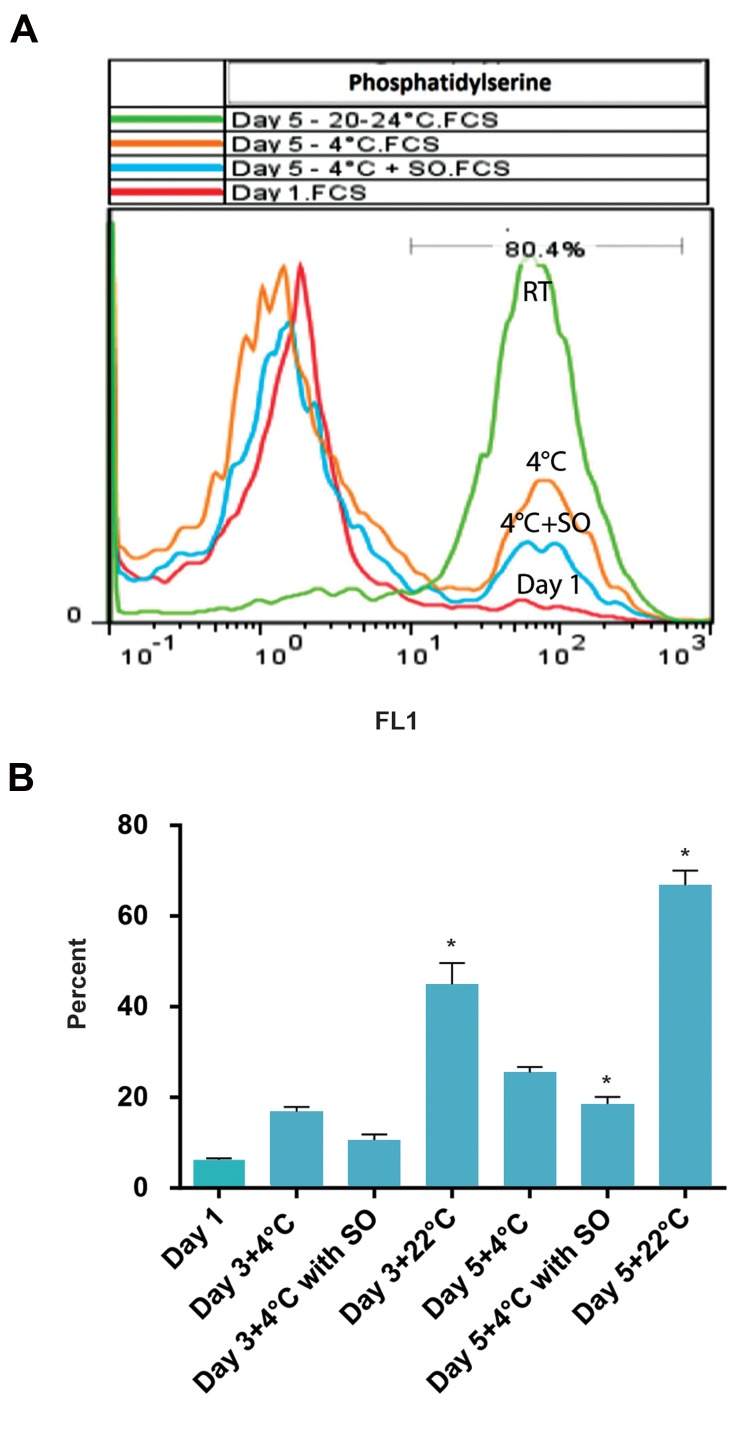
The level of phosphatidylserine (PS) exposure on platelets. A. Flow cytometry plot. PS exposure on the fifth day of storage in three groups of platelets kept at 22˚C, 4˚C and 4˚C in the presence of sodium octanoate (SO). This figure shows higher levels of PS on platelets stored at 22˚C (P<0.05). SO caused lower PS exposure on platelets at 4˚C (P<0.05) and B. PS exposure levels on platelets on different days of storage in three groups of study. The lowest exposure of PS was seen in SO-treated platelets and the highest exposure was seen in platelets stored at 22˚C. *; P<0.05.

During the storage time, the exposure of PS increased in all groups. The exposure level of PS was significantly lower in the presence of SO (4˚C) on day 5 in comparison to other groups (P<0.05). The decrease in the PS exposure level was not significantly different among the groups on the third day of storage. Nevertheless, the differences in PS exposure between the PLTs kept at 22˚C and other groups were significant (P<.05, Table 1, Fig .1).

**Table 1:** The mean and standard deviation for the study variables including phosphatidylserine, human active caspase-3 and the ingestion levels by HepG2 cells during storage days (days 1, 3 and 5) in three groups of study; platelets stored at 4˚C, 4˚C with sodium octanoate (SO) and 22˚C


Study variables n=12	Day 1	Day 3 (4˚C)	Day 3 (4˚C+SO)	Day 3 (22˚C)	Day 5 (4˚C)	Day 5 (4˚C+SO)	Day 5 (22˚C)

Phosphatidylserine (%)	6.2 ± 1.21	16.8 ± 3.7	10.57 ± 4.44	44.88 ± 16.44	25.42 ± 4.4	18.51 ± 5.59	66.77 ± 11.39
Human active caspase-3 (ng/ml)	0.647 ± 0.211	1.326 ± 0.503	1.292 ± 0.436	1.050 ± 0.418	1.485 ± 0.366	1.306 ± 0.424	1.152 ± 0.307
Hep G2 ingestion (%)	17.28 ± 2.644	28.65 ± 5.545	27.88 ± 5.458	20.24 ± 4.416	37.9 ± 2.851	30.98 ± 3.338	25.555 ± 3.161


Data are presented as mean ± SD.

### Human active caspase-3 levels in platelets

**Fig 2:**
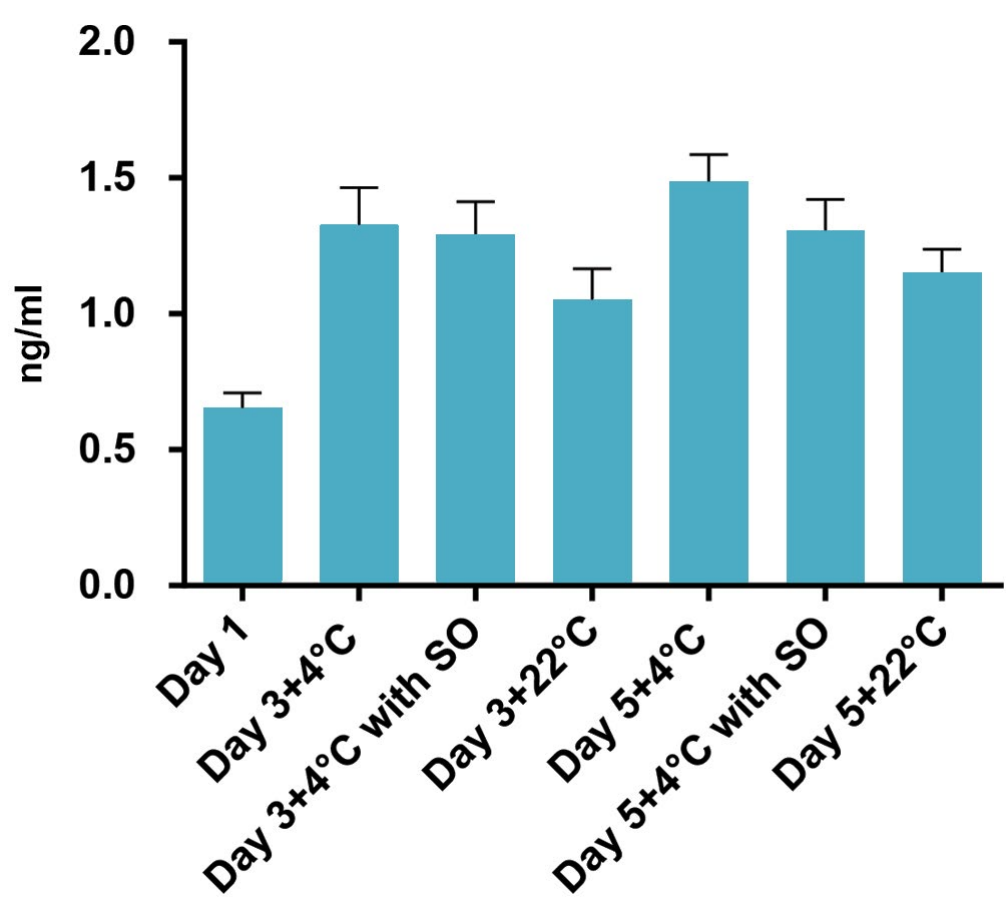
Effect of the temperature and sodium octanoate (SO) presence on the active caspase-3 levels in platelets during storage. Higher levels of active caspase-3 were observed in 4˚C- kept platelets but the presence of SO could decrease the amount of the enzyme although the difference was not significant. Lower amount of active caspase-3 was observed in platelets kept at room temperature compared to 4˚C- kept platelets.

The level of human active caspase-3 was increased in platelets during storage in all groups (Fig .2). But, a higher increase was observed in cold-stored PLTs. Although the presence of SO was accompanied by a lower increase in active caspase-3 levels in 4˚C-kept PLTs (Fig .2, Table 1), there was no significant difference in active caspase-3 levels between PLTs stored at 4˚C in the presence and absence of SO (P>0.05).

### Ingestion of the refrigerated platelets by HepG2 cells

**Fig 3:**
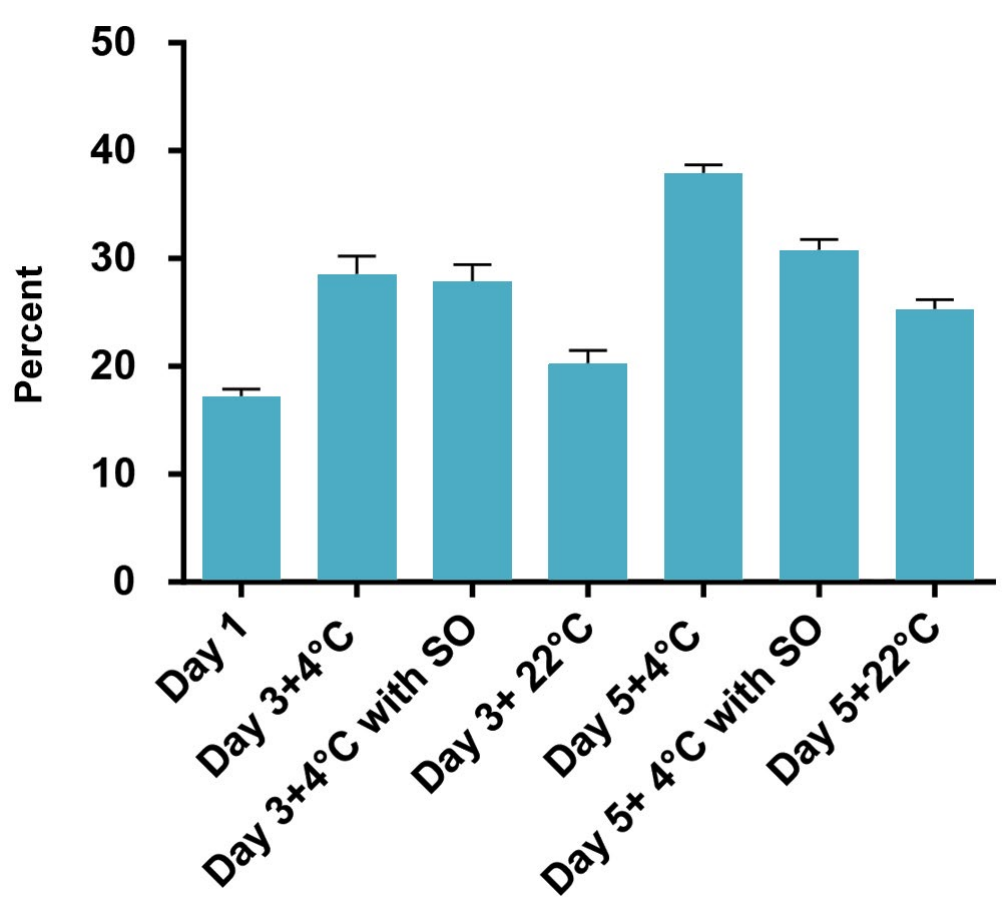
Effect of sodium octanoate (SO) on the ingestion of platelets by HepG2 cells during storage in different groups of study (22˚C, 4˚C and 4˚C+SO). As it can be seen, the ingestion rate of platelets was increased in cold. The presence of SO could decrease the clearance level although the difference was not significant. The lowest ingestion rate was observed for platelets kept at room temperature.

Storage of PLTs at 4˚C caused an increase in the ingestion rate of PLTs by HepG2 cells in comparison with 22˚C-kept PLTs during 5-day storage (P<0.05). SO caused a lower clearance rate for 4˚C-kept platelets by HepG2 cells compared to 4˚C-kept platelets in the absence of SO (Table 1, Fig .3) but the differences were not significant (P>0.05).

## Discussion

One of the strategies to reduce the complications of room temperature storage of PLTs is the maintenance of PLTs at cold temperatures. The main problem with keeping PCs in cold is the rapid removal of PLTs after transfusion due to the changes in the PLTs membrane (8). In this study, we evaluated the potential of SO to reduce the problems related to the cold storage of PLTs. For this purpose, we kept PCs for 5 days under the following three conditions: 20-22˚C, 4˚C temperature and 4˚C+SO.

In this study, the viability was reduced in all groups and the lowest level of viability was observed in PLTs stored at room temperature. It is of note that the highest level of viability was observed in PLTs treated with SO. The results of this study indicated that SO has positive effects on the survival rate of PLTs during storage at 4˚C. Our results were consistent with those reported by some other researches that showed a reduction in the survival rate of PLTs during storage due to the production of lactate and reduction of pH (23, 24).

It should be noted that this substance has not been used for protection against cold before, but it was used for protection against heat for example for stabilizing the infusible PLT membrane (20). We found that SO could reduce the levels of PS exposure and caspase-3 levels in 4˚C-stored PLTs. The ingestion rates of PLTs by HepG2 cells were also reduced in the presence of SO during storage at 4˚C. Additionally, the SO group had higher PLT count at 4˚C compared to other groups but the differences were not statistically significant. Furthermore, SO also led to improvement in PLTs survival .

Exposure of PS on the surface of PLTs is an important indicator of apoptosis. The results of our study showed that the level of PS exposure in SO-treated PLTs was lower than other groups during storage. Dasgupta et al. (24) showed that PS exposure on PLTs has a direct correlation with the activation of PLTs and the occurrence of apoptosis. Like the study of them, decreases in the exposure of PS in the SO-treated group in our study, may show the anti-apoptotic potential of this substance.

Consistent with previous studies (15-18), our study showed an increase in the amount of caspase-3 in all groups of PLTs during 5-day storage. The highest increase in caspase-3 levels in PLTs was observed at 4˚C, which may indicate the effect of cold temperature on the activation of caspase-3. In SO-treated PCs, the lower amount of caspase-3 was not significantly different from those of other groups. Our results were in line with the findings of Liu and co-workers who stated that storage in cold increases the amount of caspases in PLTs (12). Furthermore, our results were correlated with the study of Wang and co-workers who stated that the mechanism underlying the effect of medium-chain fatty acids (like octanoic acid) involves inhibition of the activities of caspase-3 and -9 in human liver cells (25).

Long-term storage of PLTs in cold leads to an increase in galactose residues on PLTs. Studies have shown that hepatocytes use the Ashwell-Morell receptor to remove these PLTs after transfusion (11). We investigated the simultaneous effect of SO and cold temperature-storage on the removal of PLTs by HepG2 cells. Studies have shown that HepG2 cells are able to remove PLTs in the culture medium and removal rate was increased after long-time storage of PLTs in cold. It has been shown that HepG2 cells do not express αMβ2 receptors. For this reason, they are not able to remove PLTs when they are stored in the cold for a short time. However, using the asialoglycoprotein receptors, HepG2 cells are capable of removing PLTs after long-time storage (26-28). Based on the results of this study, the rate of PLTs removal by HepG2 cells was increased in all groups during storage. However, the lowest increase in PLT removal was related to the 22˚C-kept PLTs, which was consistent with the findings of previous studies and was predictable. Similar to the results of previous studies, in this study, we observed an increase in PLTs removal rate during 5-day storage in cold by HepG2 cells. Despite the lower rate of PLTs’ removal by HepG2 cells in the presence of SO in comparison to 4˚C group, the differences were not significant.

## Conclusion

SO could partially moderate the effects of cold temperature on apoptosis and viability of platelets. It also decreases the ingestion rate of long-time refrigerated PLTs *in vitro*. Further studies using higher numbers of samples are required to confirm the effect of SO on reducing the clearance rate of PLTs.
